# Reliability of resting-state electrophysiology in fragile X syndrome

**DOI:** 10.1016/j.bionps.2023.100070

**Published:** 2023-07-19

**Authors:** Rui Liu, Ernest V. Pedapati, Lauren M. Schmitt, Rebecca C. Shaffer, Elizabeth G. Smith, Kelli C. Dominick, Lisa A. DeStefano, Grace Westerkamp, Paul Horn, John A. Sweeney, Craig A. Erickson

**Affiliations:** a Cincinnati Children’s Hospital Medical Center, United States; b University of Cincinnati, United States

**Keywords:** Fragile X Syndrome, Resting-state EEG, Test-retest reliability, Intraclass correlation, Electrophysiological biomarker

## Abstract

**Objective:**

Fragile X Syndrome (FXS) is the leading monogenic cause of intellectual disability and autism spectrum disorder. Currently, there are no established biomarkers for predicting and monitoring drug effects in FXS, and no approved therapies are available. Previous studies have shown electrophysiological changes in the brain using electroencephalography (EEG) in individuals with FXS and animal models. These changes may be influenced by drug therapies. In this study, we aimed to assess the reliability of resting-state EEG measures in individuals with FXS, which could potentially serve as a biomarker for drug discovery.

**Methods:**

We collected resting-state EEG data from 35 individuals with FXS participating in placebo-controlled clinical trials (23 males, 12 females; visit age mean+/−std 25.6 +/−8.3). The data were analyzed for various spectral features using intraclass correlation analysis to evaluate test-retest reliability. The intervals between EEG recordings ranged from same-day measurements to up to six weeks apart.

**Results:**

Our results showed high reliability for most spectral features, with same-day reliability exceeding 0.8. Features of interest demonstrated ICC values of 0.60 or above at longer intervals. Among the features, alpha band relative power exhibited the highest reliability.

**Conclusion:**

These findings indicate that resting-state EEG can provide consistent and reproducible measures of brain activity in individuals with FXS. This supports the potential use of EEG as an objective biomarker for evaluating the effects of new drugs in FXS.

**Significance:**

The reliable measurements obtained from power spectrum-based resting-state EEG make it a promising tool for assessing the impact of small molecule drugs in FXS.

## Introduction and background

Fragile X Syndrome (FXS) is the most common inherited genetic cause of intellectual disability and most common single gene cause of autism spectrum disorder (ASD) (Hagerman & Hagerman, 2001). FXS is caused by a CGG triplet repeat expansion (>200 CGG repeats) in the promoter region of the fragile X messenger ribonucleoprotein 1 (*FMR1*) gene on the long arm of the X chromosome. This results in gene methylation and reduction in gene transcription, with subsequent reduction of fragile X messenger ribonucleoprotein (FMRP) expression (Pieretti et al., 1991). FMRP is an RNA-binding protein that regulates synaptic function through regulation of protein translation (Maurin et al., 2018), and its deficiency results in the characteristic FXS phenotypes including developmental disability, anxiety, interfering repetitive behavior, language delay, and abnormal sensory processing (Enifeld & Hall, 1992; Kaufmann, 2002; Kaufmann et al., 1999; Loesch et al., 1993; Loesch et al., 2003; Rogers et al., 2001; Smith et al., 2016). Because FXS is an X-linked disorder, males with FXS generally present with increased impairment relative to females.

Extensive data indicate that abnormal brain electrophysiology represents the most promising translational neural indicator of brain abnormality in FXS (Ethridge et al., 2019; Jonak et al., 2020; Knoth & Lippe, 2012; Knoth et al., 2014; Lovelace et al., 2018; Pedapati et al., 2022; Smith et al., 2021;Wang et al., 2017). Cross-sectional electroencephalography (EEG) studies of FXS testing for case-control differences have documented alterations in both sensory-evoked activity and at rest (Ethridge et al., 2019; Ethridge et al., 2016; Ethridge et al., 2017; Shou et al., 2017; Smith et al., 2021; Wang et al., 2017). Our own research group has reported associations between the EEG spectral signatures in FXS and the severity of anxiety, intellectual disability, auditory attention, and social functioning impairments within FXS or its subgroups (Pedapati et al., 2022; Smith et al., 2021). High density, research-based EEG has been demonstrated to be a tolerable, noninvasive neuroimaging modality for the large proportion of individuals with FXS, most of whom cannot readily participate in MRI or other brain imaging approaches that depend on precise stability of the head. EEG abnormalities in FXS also are translationally relevant, with similar electrophysiological abnormal noted across *Fmr1* KO murine models and humans with FXS (Holley et al., 2022; Jonak et al., 2020; Kozono et al., 2020; Lovelace et al., 2018; Lovelace, Ethell, et al., 2020; Lovelace, Rais, et al., 2020; Lovelace et al., 2016; McCullagh et al., 2020; Wen et al., 2019). Importantly, preliminary data indicates that similar drug effects normalizing brain activity can be seen in mice and humans with FXS (Jonak et al., 2022).

Given these previous findings, and the lack of in vivo translational biomarkers for early phase drug testing, there is heightened interest in using high-density EEG in small molecule clinical trials in FXS to evaluate brain-activity-specific target engagement associated with drug treatment across species (Erickson et al., 2018).

The effective use of high-density EEG to detect brain activity change over time, and in response to intervention, requires a foundational demonstration that EEG signals obtained from humans with FXS remain consistent across multiple collections over the period of an early phase clinical trial. Therefore, we sought to establish the test-retest reliability of resting-state EEG data acquisition and analysis in humans with FXS using repeated measures from clinical trial settings in the placebo arm of a cross-over drug challenge study. As different arms of the trial received placebo dosing at different weeks after baseline, different individuals were followed at 2, 4 and 6 weeks.

## Method

### Resting-state EEG

**Fig. 1.**
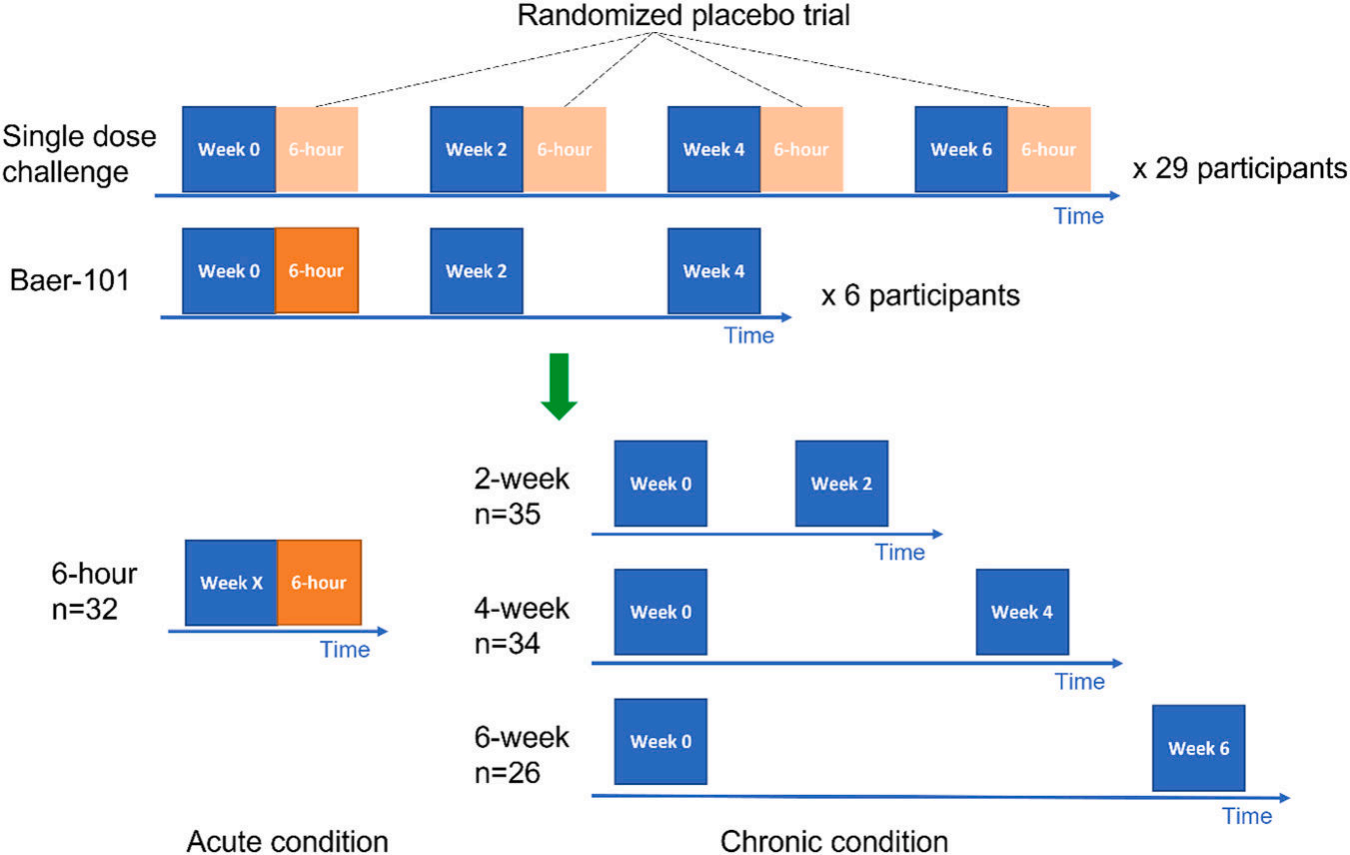
Graphical study design. Upper panel: diagram for EEG data collection from placebo trials of 2 clinical trials. Lower panel: construction of reliability testing dataset.

EEG recordings were acquired using 128-channel EGI HydroCel Geodesic Sensor Nets with a sampling rate of 1000 Hz, referenced to Cz. Five minutes of resting-state EEG was collected during each clinical trial visit with participants seated watching a silent video in a quiet environment with eyes open to facilitate cooperation, consistent with prior methodology (Pedapati et al., 2022; Wang et al., 2017). Prior to EEG data collection, trained staff familiarized participants with EEG procedures with a social story and practice net. As needed, trained staff used evidence-based behavioral techniques to reinforce sitting still and quietly, including visual timers and token boards. Resting-state EEG data during placebo treatment was utilized from two past clinical trials: 1) a single dose study comparing placebo, baclofen, acamprosate, lovastatin, and minocycline in 15–55 year old males and females with FXS (NCT02998151) with a two-week washout period between drug phases (N = 29 individual subjects; same day and 2-, 4-, and 6-week interval EEG data) and 2) a double-blind, placebo-controlled two-week treatment period with two-week washout periods (N = 6 individual subjects; same day and 2- and 4-week interval EEG data) crossover trial of two doses of AZD7325 (now BAER-101), a selective GABA A alpha 2,3 agonist, in 18–50 year old males and females with full mutation FXS (NCT03140813). For the first trial, for same day and 2-week interval data, only placebo was received by each subject between EEG recordings. For the 4- and 6-week interval data each subject would have received 1 or 2 active single study drug doses with any single active study drug dose given at least 2-weeks prior to an EEG recording. For the second trial, only placebo was received between all EEG data collections included in this analysis. Given the design of study 1 to detect same-day single drug dose potential impact on EEG signal, we included 4- and 6-week interval data expecting that a single study drug dose at least 2-weeks prior to EEG data collection would not impact resting EEG signal. A diagram of the resting-state EEG collection during the clinical trials and the construction of this test-retest dataset is shown in Fig. 1. Each clinical trial was fully reviewed and approved by the Cincinnati Children’s Hospital Medical Center Institutional Review Board and for each randomized subject, assent for participation was obtained when possible and all enrolled participants participated with written consent from their parent or guardian.

With an assumed “poor” reliability level that Intraclass Correlation (ICC) is below 0.50, we used PASS 14 (PASS 14 Power Analysis and Sample Size Software, 2015) to plan the sample size and estimate the statistical power for the reliability study. For the same day pre- and post-placebo acute condition, we determined that a sample size of 30 subjects with 2 observations per subject achieved 80% power to detect an intraclass correlation (ICC) of 0.77, assuming an ICC of 0.50 under the null hypothesis. This calculation was performed using an F-test with a significance level of 0.05. Similarly, in the chronic condition where bi-weekly replicates are compared to baseline, a sample size of 30 subjects with paired observations per subject achieved 80% power to detect an ICC of 0.75, assuming an ICC of 0.50 under the null hypothesis. In both cases, whether acute or chronic, a sample size of 30 subjects provided 80% power to declare that the ICC exceeds 0.50 at a significant level of 0.05 if the true ICC is approximately 0.75.

### EEG preprocessing

Data were blinded and coded by group, participant, and collection date. MATLAB (MATrix LABoratory, 2021) (version 2018b, The MathWorks Inc., Natick, MA, USA) was used to import EGI raw data into EEGLAB SET format. Signal was filtered with a 2 Hz highpass and 80 Hz lowpass digital zero-phase filter and a 55–65 Hz bandstop filter (with harmonics removed up to Nyquist frequency of the original sampling rate) to remove line noise using EEGLAB 14.1.2(Delorme & Makeig, 2004). Data were segmented into 2 s epochs and were inspected by a clinical research assistant (G.W.) who excluded segments containing a high amount of movement artifact and interpolated bad channels (no greater than 5% per subject) using spherical spline interpolation. Trained research staff identified independent components that reflected artifacts, e.g., eye movement and cardiac activities, to remove these features. Independent component analysis was used to perform blind source separation using the extended INFOMAX algorithm (Delorme & Makeig, 2004) with principal component analysis rank reduction (further reduced for interpolated channels).

### Derivation of EEG spectral features for analysis

**Fig. 2.**
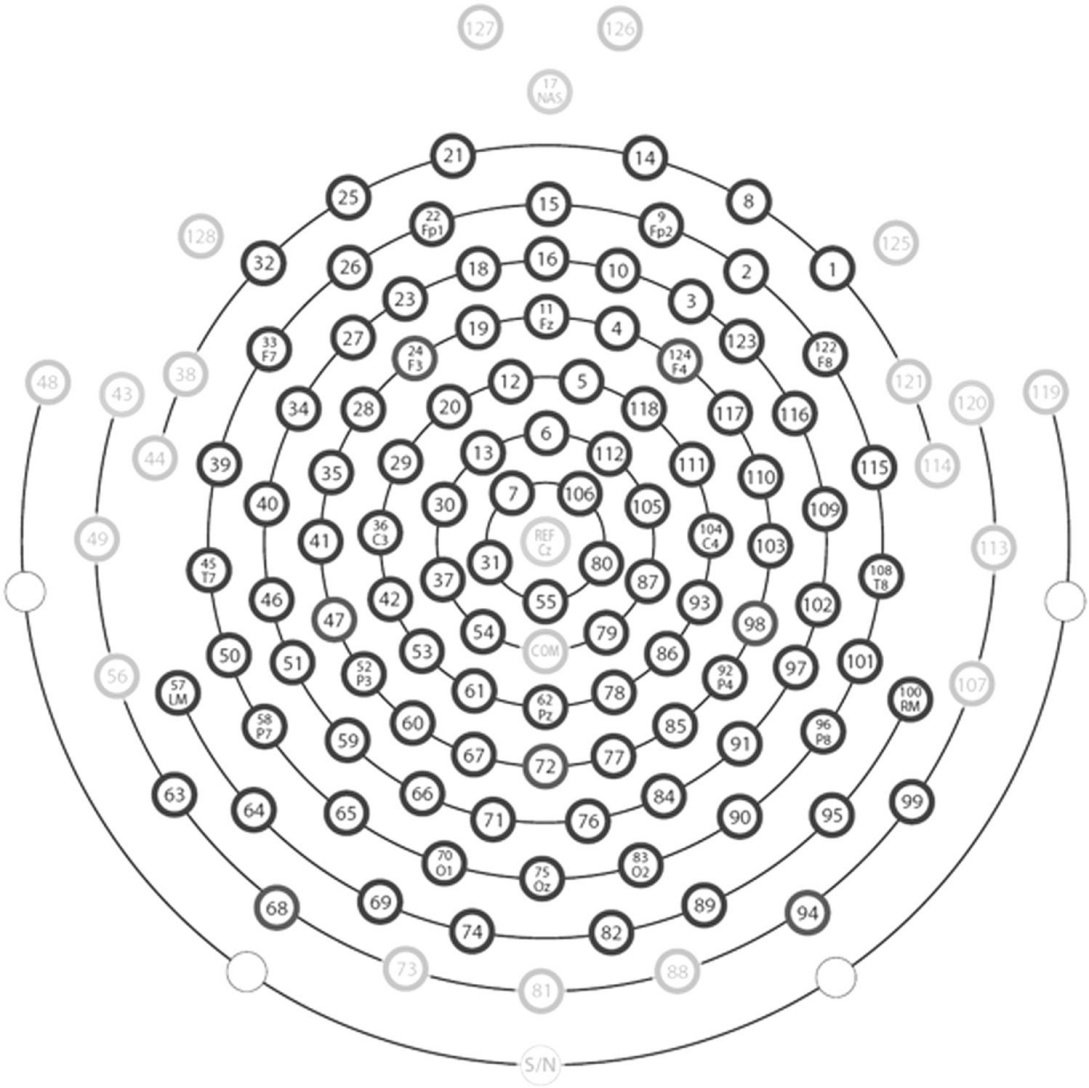
Layout of 108 selected electrodes on the EGI HydroCel Geodesic Sensor Net 128-Channel Map for whole brain average of EEG biomarkers.

Six power spectrum-based EEG biomarkers were extracted from each available EEG session for each participant: relative power across five brain frequency bands and individual peak frequency (IPF). For each EEG recording, the Welch method was applied independently to each electrode to acquire the absolute power spectrum in MATLAB (MATrix LABoratory, 2021) with a 2 s Hanning window and 50% overlap. Relative power at each frequency was calculated as the ratio between the absolute power value at that frequency and the total power between 2 and 80 Hz. Relative band power (RBP) was then calculated by averaging the relative power values within each band of interest: delta, 2–3.5 Hz; theta, 4–7.5 Hz; alpha, 8–12.5 Hz; beta, 13–30 Hz; and gamma, 30–80 Hz with a stopband at 55–65 Hz. IPF was identified as the frequency with the highest peak in the log-scaled relative power spectrum within the 5–14 Hz window (Smith et al., 2021). We averaged the feature values over 108 electrodes, excluding 20 electrodes that were located on the face and neck and are less representative of brain activity. Selected electrodes are shown as central black circles and dropped electrodes are peripheral gray circles in Fig. 2. Spectral features extraction employed the script eeg_htpCalcRestPower.m of the Cincinnati Visual High Throughput Pipeline (http://github.com/cincibrainlab).

### Reliability

Power spectrum-based EEG features were tested for reliability for each interval using intraclass correlation coefficient (ICC). The ICC model is a 2-way simple random effects model, with one factor a random sample of participants from the FXS population, and the other factor a random representation of visits from a large pool of same-condition EEG collections. As we are interested in the absolute agreement of single measurement, the suitable model is ICC(A,1) (McGraw & Wong, 1996). The corresponding sample ICC estimation applies $ICC(A,1)=\frac{BMS-EMS}{BMS+(k+1)EMS+\frac{k}{n}(RMS-EMS)}$, where k is number of visits, n is number of participants. BMS denotes the between-subject mean square, RMS denotes the within-subject between-visit mean square, and EMS denotes the residual mean square including small-valued subject-visit interaction. ICC quantifies the ratio between between-subject variability and total variability with a range in [0,1], where 0 indicates no reliability and 1 indicates perfect reliability. ICC calculation was conducted using the icc function of irr package v0.84.1 (Gamer et al., 2019) in R v4.1.0 (R: A Language and Environment for Statistical Computing, 2021).

## Results

### Participants

Among the 35 distinct participants, three individuals encountered difficulty in reaching the clinical research facility due to scheduling conflicts, financial constraints, and/or adverse weather conditions. These logistical challenges resulted in their inability to participate in the scheduled EEG sessions. Additionally, there was one participant who could not undergo the EEG assessment due to irritability, and their family decided to withdraw from participation.

Regarding successfully collected EEGs, we have 32 pairs of same day EEG data, 35 pairs of 2-week interval EEG data, 34 pairs of 4-week interval EEG data, and 26 pairs of 6-week interval EEG data. Participant age at the first EEG session, clinical evaluation scores, sex, and FXS diagnostic status using Southern Blot and PCR testing are detailed in Table 1. A total of the 11 males had mosaicism. Four males are classified as size mosaics, three as methylation mosaics, and four as size + methylation mosaics. Among our 12 female participants, two are classified as size mosaics and one as methylation mosaic. For one male and two females, only PCR testing was available, so mosaicism status is not available.

### Repeated EEG reliability evaluation

**Fig. 3.**
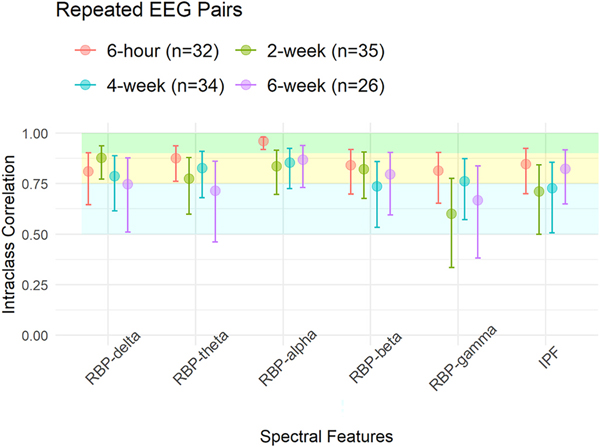
ICC and 95% CI estimations per feature and tested interval. Cyan background color range represents moderate reliability level (0.5–0.75), yellow and green are for good (0.75–0.9) and excellent reliability levels (0.9–1) (Koo & Li, 2016).

Resting-state EEG data collections at each time point had similar durations of usable data and similar number of independent components rejected in the preprocessing step (Appendix 1). Same-day reliability from resting-state EEG features were all above 0.8 (see Table 2). Longer term interval EEG data demonstrated ICC values at or above 0.6 (Table 2). Among resting-state EEG spectral features, relative power in the alpha bands demonstrated the highest ICC values (Fig. 3).

## Discussion and conclusions

This is the first report on test-retest reliability of resting state EEG measures in FXS. Given the recent demonstration of translational synchrony of resting-state EEG findings across mouse and human studies in FXS, it is a critical next step to establish that EEG findings in humans with FXS are consistent when repeated during the same day and when repeated weeks later as would be the case for a clinical trial study design. Our ICC values demonstrate that spontaneous EEG measures in adolescents and adults with FXS can be reliably reproduced. Identifying reliable, translational, and biologically based measures is critical to establish outcome measures in clinical trials for FXS. Given the behavioral and developmental challenges associated with FXS, the potential for participant characteristics to disrupt data acquisition and thus impact test-retest reliability was considered. We believe the provision of behavioral support guided by psychologists with expertise specific to FXS was critical to our success in generating reliable, reproducible EEG data in FXS.

In the context of similar resting EEG test-retest studies in humans outside of FXS, our findings in FXS generally meet or exceed the ICC values seen in other populations including typically developing adult males (Ip et al., 2018) and youth with autism spectrum disorder (ASD) (Levin et al., 2020; Webb et al., 2023). Compared to an analysis of 4 repeated EEG sessions in typically developing males, our ICC findings in FXS were comparable with the reproducibility findings across power bands, with the exception of our FXS work noting a more consistent gamma band power (ICC value range 0.37–0.52 in typically developing adult males for the upper gamma band, 45–80 Hz) (Ip et al., 2018). Similar to FXS, resting-state EEG power has been associated with clinical characteristics in ASD, including social skills, non-verbal IQ, and repetitive behavior within a male youth subgroup (Neuhaus et al., 2021). Compared to a very large recent study of youth with ASD, our FXS data indicated higher ICC values in both alpha (ASD ICC=0.730) and gamma (ASD ICC=0.555) bands, including when comparing ASD participants with co-occurring intellectual disability (ASD ICC alpha=0.725, gamma=0.476) (Webb et al., 2023). In addition, our FXS ICC values across power spectra are mostly comparable to findings from a smaller study of youth with ASD, though again with our work in FXS noting a higher ICC for IPF than that reported in ASD (ASD largest alpha peak ICC=0.62) (Levin et al., 2020). Generally, our resting EEG reproducibility findings meet or exceed the consistency of results noted in other samples including in youth with ASD, a developmental disorder known to phenotypically overlap with FXS.

It is worth noting that the ICC values we obtained for key resting EEG measures are consistent with ICC values reported for performance-based behavioral measures in FXS. These behavior measures include expressive language sampling (ELS) (Berry-Kravis et al., 2013), the Test of Attentional Performance for Children (KiTap) (Knox et al., 2012), and the Repeatable Battery of Neuropsychiatric Status (RBANS) (Berry-Kravis et al., 2006). Our findings suggest that direct brain measurements, such as EEG, hold promise for evaluating treatment outcomes in FXS. Furthermore, EEG measures may offer advantages in drug trials by potentially being less susceptible to placebo or practice effects compared to behavioral measures.

Resting-state EEG abnormalities in humans with FXS have been reproducibly established across labs (Smith et al., 2021; Van der Molen & Van der Molen, 2013; Van der Molen et al., 2014; Wang et al., 2017), are related to phenotypic features of the disorder (Smith et al., 2021; Van der Molen & Van der Molen, 2013; Van der Molen et al., 2014; Wang et al., 2017), and have shown consistent findings across murine and human study (Goswami et al., 2019; Jonak et al., 2020; Lovelace et al., 2018; Lovelace, Rais, et al., 2020; Smith et al., 2021; Van der Molen & Van der Molen, 2013; Van der Molen et al., 2014; Wang et al., 2017). Now with established test-retest reproducibility specifically in humans with FXS, resting-state EEG is well-situated to be included in small molecule and other therapeutics studies in FXS, both to aid in detection of treatment response and to identify patient subgroups who may best respond to treatment. It would also be appropriate for future studies to expand this work by evaluating test-retest reliability for other EEG metrics (e.g., network connectivity, visual and auditory induced evoked potentials) in humans in parallel with the *Fmr1* KO mouse model of FXS (Knoth et al., 2014; Schmitt et al., 2022).

The strengths of this research must be taken in the context of the limitations of this work. First, the report is limited to adolescents and adults. Studies of younger FXS participants have found EEG alterations in adolescents (Knoth et al., 2014) and age-related differences in entropy between children, adolescents, and adults (Proteau-Lemieux et al., 2021). However, there is no published data available to date describing the immediate and short-term test-retest reliability of EEG measures in younger children with FXS. Because of the age range used in our clinical trial, our sample contained almost exclusively adults, thus we did not have the sample available to examine age-related effects during prominent critical periods of brain development. Future studies focused on children with FXS are especially important, given that the impact of age on EEG measures also is not well-understood. Second, our report is limited to resting-state EEG measures. In FXS, aberrant EEG responses to auditory stimuli have been reported across species, and thus the ability to reliably reproduce EEG evoked potentials over multiple testing periods needs to be established in humans with FXS. Third, due to our sample size, it is not possible to identify potential differences in the reproducibility of resting EEG measures among subgroups of patients with FXS defined by sex, mosaicism, or level of peripheral FMRP level as measured in blood. Particularly, the difference in sample size between our male and female subgroups and the clinical heterogeneity among females weakens our ability to compare reliability based on sex. An additional weakness that could potentially lead to our underestimation of resting EEG ICC values in FXS is the fact that in study 1, for our 4- and 6-week data collection, subjects had received single doses of active study drug approximately every two weeks with at least 2 weeks having passed between last study drug dose and an EEG data point. While our study design assumed that single study drug dosing would not impact EEG signal following at least two weeks washout, it is possible carryover effects could have occurred and contributed to EEG signal change. Last, it is possible data collection, and thus ICC values, were impacted by differences in study staff within and across visits. We did our best to ensure the same study staff members were present during the same day data collection and across study visits for the same participant to minimize this effect. Despite these limitations, our data indicates resting state feature ICC values in FXS equal or higher than similar resting EEG work in other pathologies or in typically developing humans.

Our current study demonstrates for the first time the test-retest reliability of resting EEG measures in adolescents and adults with FXS. This is a critical step in identifying biomarkers that are disorder-relevant, translational across species, and demonstrate target engagement that may be useful in clinical trials. Future work must be aimed at building the capacity for reliable human EEG study in FXS beyond a small collection of tertiary care, academic large FXS centers to further enable multi-site EEG studies in FXS long-term.

## Figures and Tables

**Table 1:** Participant demographic summary.

Clinical measures: average (sd), range	Male: non-mosaic (n = 11)	Male: mosaic* (n = 11)	Female (n = 12)

Subject Age at 1st EEG session (years)	31.1 (8.5), 21.8–45.9	24.5 (7.5), 15–37	21.8 (7), 16.6–39.2
Full Scale Deviation IQ	27.2 (10.4), 11.6–44.1	47.5 (22), 19–94.1	57.9 (31.4), −10.8–91.4
ABC FXS: Irritability	11.6 (9.7), 1–32	8.3 (10.1), 0–34	9.3 (7.3), 1–20
ABC FXS: Social Withdrawal	5.1 (4.6), 0–13	6.2 (6.8), 0–24	7.3 (5.2), 0–15
ABC FXS: Stereotypy	3.8 (4), 0–12	3.6 (3.1), 0–10	3.2 (3.6), 0–13
ABC FXS: Hyperactivity	9.2 (5.1), 2–16	5.5 (5.4), 0–19	4.9 (2.5), 2–11
ABC FXS: Inappropriate Speech	5.3 (3.2), 1–10	3.5 (2.5), 0–8	3.4 (3.2), 0–8
ABC FXS: Social Avoidance	1.8 (1.8), 0–5	4.2 (2.6), 0–9	5.4 (4.8), 0–12

EEG Session Count per Tested interval	Male: non-mosaic (n = 11)	Male: mosaic (n = 11)	Female (n = 12)

Same day (4–6 h) interval	N = 11	N = 10	N = 10
2-week interval	N = 11	N = 11	N = 12
4-week interval	N = 11	N = 10	N = 12
6-week interval	N = 10	N=8	N=7

* Mosaic status refers to either CGG repeat size mosaicism or methylation mosaicism. For one participant, only PCR testing was available (i.e., “Mosaicism unknown”).

**Table 2:** Sample ICC estimates^11^ listed by EEG features and time intervals, using single-measure, absolute-agreement, 2-way simple random-effects model.

Intervals Features	4–6 h (same day) (n = 32)	2-week (n = 35)	4-week (n = 34)	6-week (n = 26)

RBP-delta	0.810	0.878	0.786	0.746
RBP-theta	0.875	0.774	0.826	0.714
RBP-alpha	0.959	0.836	0.853	0.868
RBP-beta	0.840	0.822	0.737	0.796
RBP-gamma	0.814	0.600	0.761	0.668
IPF	0.847	0.710	0.727	0.824

**Fig. A.1.**
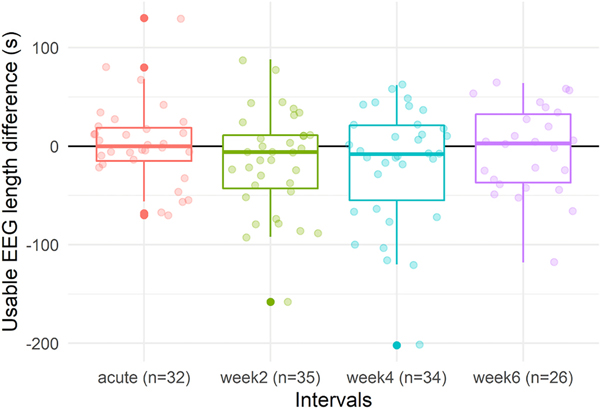
Group-level (boxplot) and subject-level (scatter plot) difference from baseline on usable EEG length for 4 tested intervals.

**Fig. A.2.**
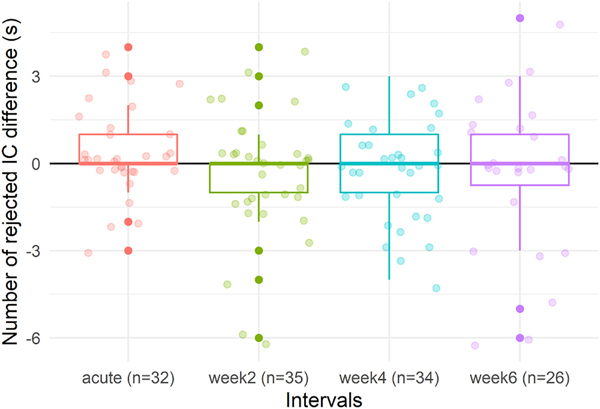
Difference from baseline on number of rejected ICs for 4 tested intervals. Due to the ties (identical values) cross subjects, the 25% percentile and the 50% percentile overlap for same day 4–6 h interval, the 50% percentile and the 75% percentile overlap for 2-week interval.

**Fig. A.3.**
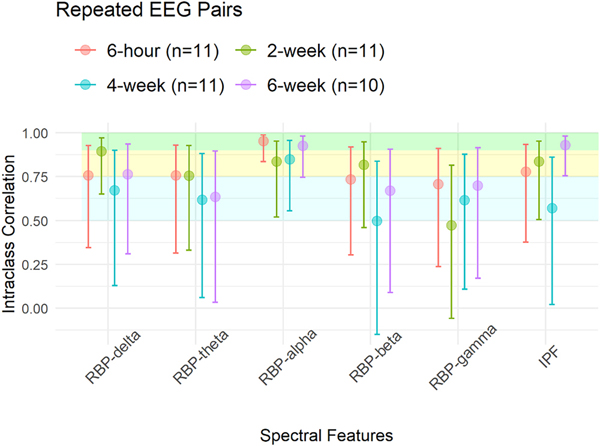
ICC and 95% CI estimations per feature and tested interval for full mutation fully methylated males (n = 11). Cyan background color range represents moderate reliability level (0.5–0.75), yellow and green are for good (0.75–0.9) and excellent reliability levels (0.9–1).

**Fig. A.4.**
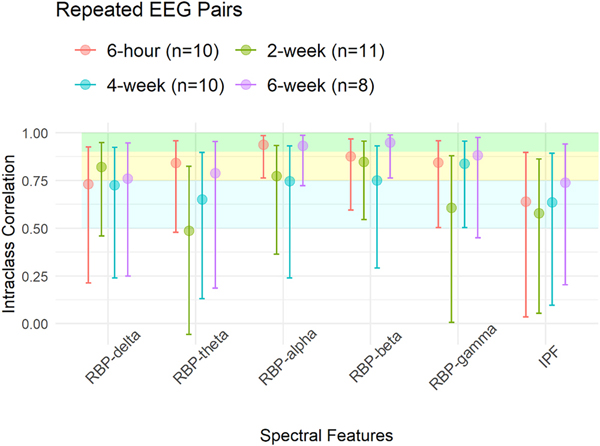
ICC and 95% CI estimations per feature and tested interval for mosaic males (n = 11). Cyan background color range represents moderate reliability level (0.5–0.75), yellow and green are for good (0.75–0.9) and excellent reliability levels (0.9–1).

**Fig. A.5.**
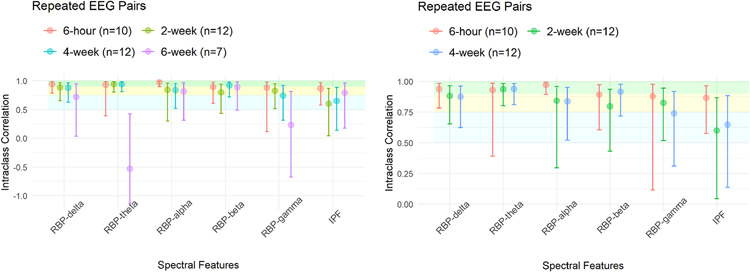
ICC and 95% CI estimations per feature and tested interval for females (n = 12). Cyan background color range represents moderate reliability level (0.5–0.75), yellow and green are for good (0.75–0.9) and excellent reliability levels (0.9–1). Left panel: ICC evaluation for all 4 intervals. Right panel: ICC evaluation excluding 6-week interval in which 7 available subjects showed lack of heterogeneity.

## Footnotes

1 Our primary analysis focuses on direct sample ICC estimates, ICC 95% confidence interval estimates for population are marked in Fig. 3

## Appendix 1. – EEG data quality cross visits

**Table A.1:** Usable resting EEG duration summary table.

	Timepoint	n	Average + /− SD (seconds)	Range (seconds)

Acute (same day) interval	Pre-placebo	32	211.2 + /− 63.9	92–284
	Post-placebo	32	213.9 + /− 63.4	58–306
2-week intervals data	Baseline	35	221.0 + /− 55.7	94–288
	Week 2	35	205.2 + /− 67.4	70–334
	Week 4	34	205.1 + /− 75.7	46–282
	Week 6	26	240.8 + /− 54.5	40–320

**Table A.2:** Number of rejected Independent Components (IC) summary table.

	Timepoint	n	Average + /− SD (count)	Range (count)

Acute (same day) interval	Pre-placebo	32	10.7 + /− 1.65	6–12
	Post-placebo	32	11.1 + /− 1.48	8–13
2-week intervals data	Baseline	35	11.0 + /− 1.67	5–12
	Week 2	35	10.6 + /− 1.94	3–12
	Week 4	34	10.8 + /− 1.79	6–13
	Week 6	26	10.5 + /− 2.08	6–13
